# C-Reactive Protein as a Peripheral Biomarker in Schizophrenia. An Updated Systematic Review

**DOI:** 10.3389/fpsyt.2018.00392

**Published:** 2018-08-23

**Authors:** Guillaume Fond, Christophe Lançon, Pascal Auquier, Laurent Boyer

**Affiliations:** EA 3279, CEReSS-Health Service Research and Quality of Life Center, School of Medicine-La Timone Medical, Aix-Marseille Université, AP-HM Assistance Publique des Hôpitaux de Marseille, Marseille, France

**Keywords:** C-reactive protein (CRP), schizophrenia, peripheral biomarker, onset risk, cognition, physical health, nicotine dependence

## Abstract

**Objectives:** The objective of this systematic review was to synthetize the published data on the relationships between elevated blood C-reactive protein (CRP) levels and schizophrenia (SZ) onset risk, illness characteristics and treatments, cognition and physical health.

**Method:** The systematic bibliographic searches have been carried out according to the Cochrane methodology. Medline, web of science, Google Scholar with each database being searched from inception to November 2017.

**Results:** 53 studies were included in the present review. While meta-analyses including case-control studies suggest a clear association between CRP and SZ, one other study has suggested that CRP-associated genes were associated with a lower risk of SZ onset. Increased CRP has been significantly associated with positive symptoms in acute phase psychosis, while studies including community-dwelling stabilized subjects did not find such an association. Abnormal CRP has been associated with a wide range of cognitive impairment in SZ stabilized individuals. Body Mass index has been extensively associated with increased CRP in SZ subjects; and increased CRP has been identified as a risk factor for metabolic syndrome and cardiovascular risk in SZ subjects. Increased CRP has also been associated with high nicotine dependence in SZ smokers and one study has suggested that increased CRP was associated with sedentary behavior.

**Conclusion:** In the light of the above-mentioned studies, increased hs-CRP may be reasonably suggested as a marker for SZ onset risk, as well as a risk factor for increased positive symptoms, cognitive impairment, hypovitaminosis D, microbiota disturbances, cardiovascular and metabolic syndrome risk in SZ subjects, and increased nicotine dependence in SZ smokers. In case of increased CRP levels, anti-inflammatory strategies (add-on anti-inflammatory drugs including aspirin and omega 3 fatty acids, vitamin D supplementation, physical activity, probiotics) should be also further evaluated.

**Limits:** Most of the studies were cross-sectional and cohort studies are needed to determine the temporal relationship between increased CRP and the psychiatric outcomes.

## Introduction

Inflammation is a complex physiologic response to injury or tissue destruction, and involves recruitment of immune cells. The inflammatory hypothesis of major psychiatric disorders, posits that inflammatory processes are involved in the pathogenesis of psychiatric conditions and may underpin some of their neurobiological correlates (1). SZ is the psychiatric disorder causing the most severe burden of illness for the individual, by its significant cognitive and social functioning impairments but also due to its medical comorbidities (2). A bundle of studies have suggested a link between inflammation and at least a subgroup of SZ subjects (1, 3–5).

C-reactive protein (CRP) is the most commonly used biomarker of systemic inflammation worldwide. CRP is a standard laboratory exam and can be measured in the peripheral blood and analyzed in any clinical laboratory around the globe. CRP is therefore a very attractive potential clinical biomarker in psychiatric disorders (6). CRP is an acute phase protein that is produced by hepatocytes. The high-sensitivity CRP (hs-CRP) assay has a lower limit of detection of 0.1 mg/L. The measurement of CRP is useful in the diagnosis and monitoring of many acute and chronic inflammatory conditions, including obesity and the metabolic syndrome, which can cause elevations of hs-CRP in the range of 3–10 mg/L (7). Increased CRP has been recently associated with cognitive impairment in SZ, which suggests that anti-inflammatory strategies may improve the debilitating course of this illness (8).

The objective of the present systematic review was to determine if dosing CRP blood levels was relevant to synthetize cross-sectional studies and to determine (i) SZ onset risk in general population (ii) clinical characteristics and treatments (iii) cognitive deficits and (iv) physical health in SZ in- and outpatients.

## Materials and methods

This meta-analysis is based on the PRISMA criteria (Preferred Reporting Items for Systematic reviews and Meta-Analysis). The systematic bibliographic searches have been carried out according to the Cochrane methodology. These were performed to find relevant English and non-English language trials from the following databases: Medline, web of science, Google Scholar with each database being searched from inception to November 2017. Medline is considered as the database of highest quality level, and google scholar as the database with the largest referencing of studies. Most of the international congress posters and abstracts are referenced in web of science. Altogether, these three searches enable a comprehensive exploration and limit the risk of publication bias. The associated articles were also explored, to limit the risk of bias associated with the search terms. The primary search strategy was “C-reactive protein” or “CRP” and “schizophrenia” or “ultra high risk psychosis.” Two reviewers (GF and LB) decided on eligibility and extracted data from included studies. The design of the studies, data extraction and data synthesis are described in Table 1. Only studies including at least a subgroup of SZ patients or at risk for SZ onset were included in the present review. As this meta-analysis mainly involved data from published studies, an institutional review board approval was not required.

**Table 1:** Studies exploring relationships between C-reactive protein (CRP) blood levels and schizophrenia (SZ): designs and major findings.

**Authors**	**Year**	**Design**	**Population**	**Major outcomes**
**SZ ONSET RISK (*****N*** = **13)**				
Metcalf	2017	Prospective	6,362 HC 15–16 years adolescents	Using CRP as a categorical variable, those with high (>3 mg/L) compared with low (<1 mg/L) CRP levels at baseline were more likely to develop SZ; adjusted OR 4.25 (95% CI, 1.30–13.93).
Prins	2016	Genetic cross-sectional study	>25,000 SZ >30,000 controls from populations of European ancestry	Genetically elevated CRP levels showed a significant potentially protective causal relationship with SZ risk.
Inoshita	2016	Control study and meta-analysis	418 SZ	Serum CRP levels were significantly higher in SZ patients than in the controls.
Aymaropoulos	2015	Case-control	460 SZ 241 HC	CRP levels were significantly higher in SZ subjects but smoking and BMI were not controlled.
Khandaker	2014	Prospective cohort study	4,500 children	While higher levels of the systemic inflammatory marker IL-6 in childhood are associated with an increased risk of developing depression and psychosis in young adulthood, the results were non-significant for CRP.
Wium-Andersen	2014	Prospective cohort study	78,810 HC	Baseline elevated plasma CRP was associated with a 6- to 11-fold increased risk of late- and very-late-onset schizophrenia in the general population.
Joshi	2014	Case-control	45 SZ 41 HC	The Schizophrenia subjects showed statistically significant increased hs-CRP values.
Dickerson	2013	Case-control	295 SZ 228 HC	The individuals with schizophrenia had significantly increased odds of having elevated levels of CRP relative to both the 75th and 90th percentile levels of the controls after adjustment for age, gender, race, maternal education, smoking status, and BMI.
Lin	2013	Case-control	36 SZ 36 matched HC	Ancova adjusted for age and BMI revealed a significant increase in the hsCRP levels in the schizophrenic group (1.4 mg/L, SD = 1.5 mg/L) in comparison with the control group (0.9 mg/L, SD = 1.4 mg/L; *P* = 0.013).
Fawzi	2011	Case-control	200 SZ antipsychotic-free 200 HC	In Egyptian men, waist circumference and SZ diagnosis were strong predictors of raised CRP levels independently of a number of potentially confounding variables. In antipsychotic-free SZ patients, CRP level was higher than in HC and is positively correlated with negative symptomatology as measured by the PANSS.
Suvisaai	2011	Case-control	45 SZ 57 ONAP 37 affective psychosis matched controls	SZ subjects had significantly higher CRP blood levels. CRP was influenced by both antipsychotic medication and nonaffective psychosis.
Zakharyan	2010	Case-control genetic	103 SZ 105 HC	None of the CRP rs1417938, rs1800947, rs1205 variants was associated with schizophrenia.
Hope	2009	Case-control	186 SZ 244 HC	There were no differences in CRP blood levels between the groups.
**CLINICAL CHARACTERISTICS AND TREATMENTS (*****N*** = **19)**				
Aas	2017	Case-control	148 SZ and 123 BD vs. 212 HC	Patients had increased levels of hs-CRP (*P* < 0.001, Cohens *d* = 0.4). The severity of childhood abuse (up to three types of abuse: sexual abuse, physical abuse, and emotional abuse) was associated with higher hs-CRP blood levels (*f* = 5.47, *P* = 0.001, Cohen's *d* = 0.3). Combined effects of patient status and severity of childhood abuse were found for elevated hs-CRP (*f* = 4.76, *P* < 0.001, Cohen's *d* = 0.4). Differences among the groups disappeared when BMI was added to the model.
Hartwig	2017	Two-sample mendelian randomization	>30,000 SZ >45,000 HC	The pooled odds ratio estimate using 18 CRP genetic instruments was 0.90 (random effects 95% CI, 0.84–0.97; *P* = 0.005) per 2-fold increment in CRP levels.
Wang	2017	Meta-analysis	1,963 SZ 3,683 HC	Compared with non-SZs, blood CRP levels were moderately increased in SZ (SMD 0.53, 95% CI 0.30–0.76) irrespectively of study region, sample size of included studies, patient mean age, age of SZ onset and patient body mass index. Patients in Asia or Africa (*n* = 6, SMD 0.73, 95% CI 0.26–1.21) and whose age <30 years (*n* = 5, SMD 0.76, 95% CI 0.07–1.58) had substantially higher CRP levels.
Christiano	2017	Cross-sectional	35 SZ	CRP levels were higher in cases with greater disease severity.
Frydecka	2015	Case-control	151 SZ 154 HC	hsCRP were higher in SZ subjects compared to HC. hsCRP levels were associated with insidious psychosis onset, duration of illness and chronic schizophrenia course with deterioration.
Devaranayanan	2017	Case-control	40 SZ 40 HC	Hs-CRP levels were not associated with the disease severity.
Faugere	2017	Cross-sectional	307 SZ	In multivariate analyses, patients with abnormal CRP levels [>3 mg/L, *N* = 12 (40.4%)] were found to have higher depression scores than those with normal CRP levels in multivariate analyses (*p* = 0.035, OR = 1.067, 95% CI = 1.004–1.132). No significant association between CRP levels and antidepressant consumption was found.
Fond	2016	Cross-sectional	219 SZ	Overall, 43 (20.1%) of the subjects received a diagnosis of comorbid current depression, and 51 (31.9%) had ongoing antidepressant treatment. Abnormal CRP levels in schizophrenia [>3 mg/L, *N* = 63 (28.8%)] were found to be associated with antidepressant consumption, but not with depression. In a multivariate model, abnormal CRP was associated with antidepressant consumption (aOR 2.8, 95%CI 1.22–6.62). Metabolic syndrome was also independently associated with abnormal CRP (aOR2.6, 95%CI 1.01–6.71).
Barzilay	2016	Cross-sectional	213 SZ	Inpatients with elevated CRP (>1 mg/L) displayed increased aggressive behavior compared to patients with normal CRP levels.
Joseph	2015	Case-control	88 SZ 71 HC	hs-CRP levels were significantly higher in individuals with SZ than in comparison subjects. Higher hs-CRP levels in the SZ group were associated with female gender, more severe negative symptoms, greater medical comorbidity, and worse metabolic risk factors including BMI, fasting glucose, and hemoglobin A1c levels. hs-CRP was not related to age, race, education, smoking status, antipsychotic dosage, or cognitive impairment.
Fernandes	2016	Meta-analysis (26 studies)	>85,000 subjects	CRP levels were moderately increased in persons with SZ regardless of the use of antipsychotics and did not change between the first episode of psychosis and with progression of SZ (*g* = 0.66, 95% confidence interval (95% CI) 0.43–0.88, *P* < 0.001, 24 between-group comparisons, *n* = 82,962). The extent of the increase in peripheral CRP levels paralleled the increase in severity of positive symptoms, but was unrelated to the severity of negative symptoms. CRP levels were also aligned with an increased BMI. Conversely, higher age correlated with a smaller difference in CRP levels between persons with SZ and controls. Furthermore, CRP levels did not increase after initiation of antipsychotic medication notwithstanding whether these were typical or atypical antipsychotics (*g* = 0.01, 95% CI −0.20 to 0.22, *P* = 0.803, 8 within within-group comparisons, *n* = 713).
Faugere	2015	Cross-sectional	256 SZ	After adjusting for key socio-demographic and clinical confounding factors, patients with high levels of CRP (>3 mg/L) had a lower QoL than patients with normal CRP levels (OR = 0.97, 95% CI = 0.94–0.99). An investigation of the dimensions of QoL revealed that psychological well-being, physical well-being and sentimental life were the most salient features of QoL associated with CRP. Significant associations were found between lower educational level (OR = 4.15, 95% CI = 1.55–11.07), higher BMI (OR = 1.16, 95% CI = 1.06–1.28), higher Fagerström score (OR = 1.22, 95% CI = 1.01–1.47) and high levels of CRP.
Sobis	2015	Interventional	17 SZ	After 28 days of aripiprazole treatment a significant reduction in hsCRP has been detected (*p* < 0.001).
Micoulaud-Franchi	2015	Cross-sectional	55 SZ outpatients	Abnormal CRP [>3 mg/L, *N* = 15 (27.3%)] was associated with higher rate of sensory gating deficit (60 vs. 12.5%, *p* < 0.001).
Wyzokinski	2015	Cross-sectional	485 SZ	Increased CRP level (>3 mg/L, 35.7%) was associated with age and female gender.
Meyer	2009	3 months Follow-up interventional (CATIE study)	789 SZ	There were significant treatment differences in CRP at 3 months of antipsychotic treatment, with a differential impact of baseline values. In overall comparisons, quetiapine and olanzapine had the highest median levels for CRP. In those with low baseline CRP (< 1 mg/L), olanzapine was significantly different than perphenazine (*p* < 0.001), risperidone (*p* < 0.001), and ziprasidone (*p* = 0.002) for CRP. The 18-months repeated measures CRP analysis confirmed the significantly higher values for olanzapine in those with low baseline CRP.
Akanji	2009	Case-control	207 SZ 165 HC	SZ subjects had significantly greater serum concentrations of hsCRP. There were significant associations between hsCRP and (i) age in both groups; (ii) BMI in HC but not in SZ. In the latter, hsCRP levels were: (a) marginally higher in women with later age of disease onset; (ii) highest with remission and with catatonic features; and (iii) lower with family history of psychosis.
Carrizo	2008	Case-control	88 SZ 34 first-degree relatives	The typical AP group had the highest CRP level (*p* = 0.013) in spite of having the lowest BMI. Patients as a single group had higher CRP levels than relatives (*p* = 0.003).
Baptista	2007	16 weeks follow-up Interventional	60 SZ inpatients with chronic severe illness	CRP levels significantly increased after olanzapine switch as well as metabolic markers.
Fan	2007	Cross-sectional	26 SZ	Subjects with CRP >5 mg/L (*N* = 5) scored significantly higher on the PANSS total score, negative symptom subscale score and general psychopathology subscale score.
**COGNITION (*****N*** = **8)**				
Dorofeikova	2017	Cross-sectional	125 SZ inpatients	Thought disorders were more pronounced in patients with CRP levels >3 mg/L [*N* = 26 (21.4%)] (*r* = 0.433, *p* = 0.017). Increased CRP was also found in more aggressive, agitated patients (*r* = 0.394, *p* = 0.031). Patients with a smaller volume of retention of short-term memory were characterized by higher CRP levels (*r* = −0.280, *p* = 0.045).
Bulzacka	2016	Cross-sectional	369 SZ outpatients	Multiple factor analysis revealed that abnormal CRP levels [>3 mg/L, *N* = 104 (28.2%)] were associated with impaired General Intellectual Ability and Abstract Reasoning (aOR = 0.56, 95%IC 0.35–0.90, *p* = 0.014), independently of age, sex, education level, psychotic symptomatology, treatments and addiction comorbidities. Abnormal CRP levels were also associated with the decline of all components of working memory (respectively effect size (ES) = 0.25, *p* = 0.033, ES = 0.27, *p* = 0.04, ES = 0.33, *p* = 0.006, and ES = 0.38, *p* = 0.004) and a wide range of other impaired cognitive functions, including memory (ES = 0.26, *p* = 0.026), learning abilities (ES = 0.28, *p* = 0.035), semantic memory (ES = 0.26, *p* = 0.026), mental flexibility (ES = 0.26, *p* = 0.044), visual attention (ES = 0.23, *p* = 0.004) and speed of processing (ES = 0.23, *p* = 0.043).
Johnsen	2016	interventional	124 SZ inpatients at admittance	There was an inverse relationship between overall cognitive performance and CRP level at admittance.
Dickerson	2013	Case-control	295 SZ outpatients	There was an inverse relationship between CRP levels and performance on RBANS total (*t* = −2.48, *p* = 0.015); RBANS immediate memory (*t* = −2.16, *p* = 0.033); RBANS attention (*t* = −2.18, *p* = 0.032); RBANS language (*t* = −2.13, *p* = 0.036); Trail Making A (*t* = −2.39, *p* = 0.019).
Garcia-rizo	2012	Cross-sectional	62 antipsychotic-naïve SZ patients	CRP levels were significantly higher in the deficit patients (3 vs.2 mg/l).
Dickerson	2012	Cross-sectional	413 SZ outpatients	The risks of decreased cognitive functioning associated with HSV-1 exposure and elevated levels of CRP were independent and additive. There was no effect of HSV-1 exposure and CRP levels on the severity of symptoms as measured by the PANSS (all *p* > 0.5).
Dickerson	2007	Cross-sectional	413 SZ outpatients	Elevated serum levels of C-reactive protein in schizophrenia are associated with the severity of cognitive impairment but not of psychiatric symptoms.
**PHYSICAL HEALTH (*****N*** = **13)**				
Horsdal	2017	Cross-cohort	17,314	Elevated CRP levels were associated with increased all-cause mortality by adjusted HRs of 1.56 (95% CI: 1.02–2.38) for levels 3–10 mg/L and 2.07 (95% CI: 1.30–3.29) for levels above 10 mg/L compared to individuals with levels below 3 mg/L.
Fond	2017	Cross-sectional	345 SZ	CRP levels ≥ 3 mg/L were associated with severe nicotine dependence (29 vs. 15%, OR = 2.8, *p* = 0.003) and BMI (OR = 1.1, *p* < 0.0001), independently of socio-demographic characteristics and antidepressant intake.
Lally	2016	Cross-sectional	324 SZ outpatients	Accounting for age, gender, ethnicity and season of sampling, serum 25-OHD levels were negatively correlated with waist circumference (*r* = −0.220, *p* < 0.002), triglycerides (*r* = −0.160, *p* = 0.024), total cholesterol (*r* = −0.144, *p* = 0.043), fasting glucose (*r* = −0.191, *p* = 0.007), HbA1c (*r* = −0.183, *p* = 0.01), and serum CRP levels (*r* = −0.211, *p* = 0.003) and were linked to the presence of metabolic syndrome.
Stubbs	2015	Cross-sectional	250 SZ	Higher sedentary behavior (β = 0.155, *p* = 0.01), female gender (β = 0.229, *p* = 0.001), waist circumference (β = 0.205, *p* = 0.003) and non-white ethnicity (β = 0.181, *p* = 0.005) was associated with elevated CRP levels [>5 mg/L, *N* = 91 (36.4%)] after adjustment for confounding variables.
Popovic	2015	Cross-sectional	93 SZ long-term inpatients	Elevated CRP was identified as a predictor of metabolic syndrome independently of diabetes mellitus in family history, BMI > 25 kg/m^2^, and hyperlipidemia in family history (*p* = 0.042).
Mori	2015	Interventional (CATIE study)	1,450 SZ	After controlling for potential confounders, blood CRP, interleukin-6, and leptin were significant predictors of all five individual components of the metabolic syndrome (as both continuous and categorical outcome measures).
Zhu	2015	Cross-sectional	93 SZ 93 family-matched HC	Mean levels of CRP and 25(OH)D were 43.3% higher and 26.7% lower for patients compared to controls, respectively. 25(OH)D were inversely associated with CRP in the patients, but not in the controls.
Fawzi	2015	Cross-sectional	100 SZ	In a multiple regression analysis, total energy intake and BMI emerged as the main independent predictors of deterioration in most inflammatory and psychopathology parameters.
Klemettila	2014	Cross-sectional	190 resistant SZ	hs-CRP was associated with obesity after controlling for age and smoking.
Severance	2013	Cross-sectional	141 SZ 78 HC	The serological surrogate markers of bacterial translocation (soluble CD14 and lipopolysaccharide binding protein) were both significantly correlated with CRP [*R*(2) = 0.26–0.27, *p* < 0.0001] and elevated in females compared to males (*p* < 0.01).
Vuksan-Cusa	2013	Cross-sectional	60 BD+ 62 SZ 59 HC	In the patient group, CRP was correlated with waist circumference and diastolic blood pressure. Elevated CRP was not a significant predictor of MetS (*p* > 0.05).
Dieset	2012	Cross-sectional	361 SZ	After adjusting for confounders: BMI, triglycerides and glucose were associated with increased hsCRP (*p* = 0.041–0.001). In patients treated with SGA, elevated hsCRP was significantly associated with high BMI (*p* = 0.012), and with high glucose levels (*p* = 0.003).
Sicras-Mainar	2013	Cross-sectional	705 SZ spectrum disorder	After adjusting for age, gender, smoking and presence of neoplasm or inflammatory diseases, CRP was linearly associated with 10-years CVD risk stratified by risk (low, moderate, high/very high): respectively, 2.3 (95% CI: 2.1–2.5), 3.1 (2.6–3.5) and 3.7 (3.2–4.1) mg/L; *F* = 13.5, *P* < 0.001. Patients with known CVD also showed higher CRP levels: 3.7 (2.9–4.5) vs. 2.5 (2.4–2.7) mg/L, *P* = 0.008; and higher probability of above-normal values; odds ratio = 4.71 (2.01–11.04), *P* < 0.001.
Vuksan-Cusa	2010	Cross-sectional	63 SZ	The prevalence of the MetS was 37%. CRP > 5 mg/L was significantly associated with the presence of MetS.

*BD, bipolar disorder; ONAP, other non-affective psychoses; SGA, second generation antipsychotic; MetS, metabolic syndrome; BMI, body mass index*.

## Results

**Figure 1:**
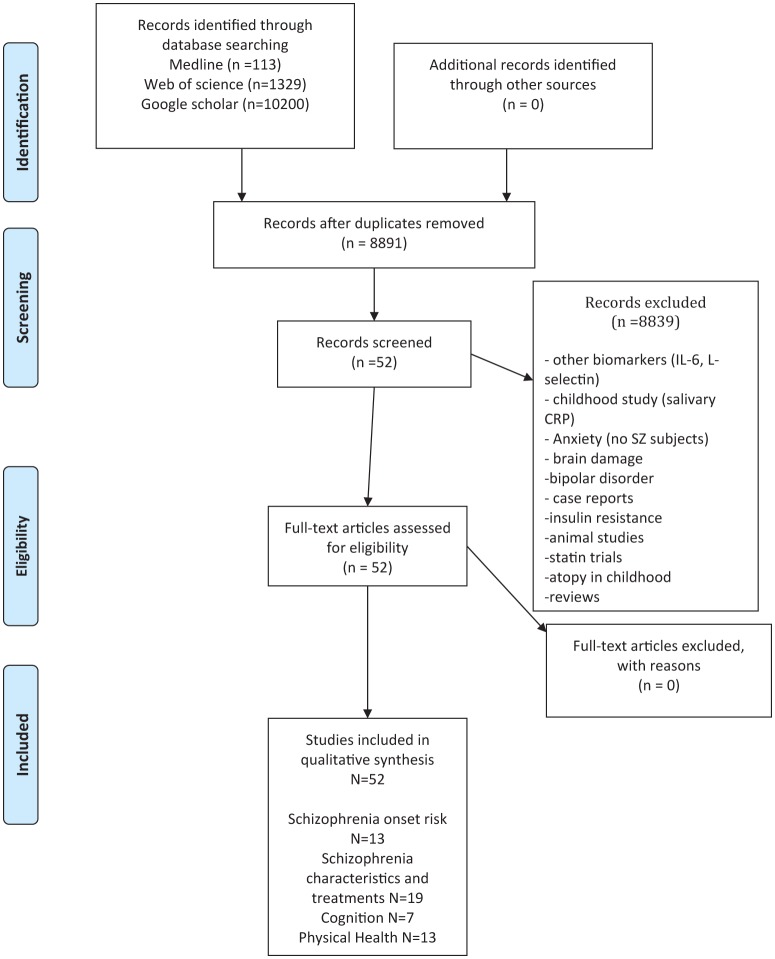
Study selection process (PRISMA flow diagram).

The study selection process is presented in Figure 1. Overall, 53 studies were included in the final qualitative analysis. The major findings and design characteristics of the included studies are presented in Table 1.

### Schizophrenia onset risk

A recent meta-analysis including 18 studies (1,989 SZ vs. 3,689 healthy controls) has concluded that higher CRP levels were associated with increased risk of SZ, especially for young adult patients < 30 years and independently of body mass index (9). Another recent meta-analysis has concluded that the association between elevated CRP and SZ was robust (1). These meta-analyses underlined that the difference between CRP levels of SZ subjects and HC were higher in young people (aged < 30), due to an increase of CRP in the HC group in older subjects and a stable increased CRP levels in SZ throughout the illness course, independently of administered treatments (1, 9).

A prospective study including 6,362 healthy adolescents ages 15–16 years has found that elevated CRP levels were predictive of SZ onset at age 27 (10). Elevated CRP in childhood has not been identified as predictive of psychotic disorder or SZ onset in adolescence in one prospective study including 4,500 children (11). One population-based prospective study has reported that elevated plasma CRP levels were associated with late-onset SZ (12).

Inconsistently with the previous findings, a large genetic study including >25,000 SZ subjects and >30,000 controls has concluded that alleles associated with increased CRP were protective from schizophrenia with a moderate effect (13).

### Clinical characteristics and treatments

One recent meta-analysis has concluded that increased CRP levels were associated with positive but not negative symptoms in schizophrenia (1). However, many studies have reported no association between abnormal CRP levels and psychotic symptomatology (14–18). In one case control study, hsCRP levels were associated with insidious psychosis onset, duration of illness and chronic schizophrenia course with deterioration (17).

Higher CRP levels have been found in SZ females in some studies (19–21) but not all (15, 22, 23). This association has not been explored in the meta-analysis of Fernandes et al. (1).

One study has found an association between current depression and abnormal CRP in SZ subjects (24) while another has found no association with depression but with antidepressant consumption (16). One study has found that history of childhood trauma was associated with increased CRP in SZ adulthood (25).

Abnormal CRP levels have been further associated with increased nicotine dependence in two studies (15, 26), but not with daily tobacco smoking (23). Abnormal CRP levels have been associated with increased aggressive behavior in SZ inpatients in one study (27) but this has not been replicated in one other SZ population (23). Increased CRP levels have been associated with impaired sensory gating in one small sample of stabilized SZ individuals (18).

The mean levels of CRP have not been found to change across the progression of the illness in one recent meta-analysis (1). While this meta-analysis has suggested that the antipsychotic treatment onset or modification did not impact mean peripheral CRP levels, one recent study has found that some specific psychotropic drugs were found to be associated with increased CRP levels (especially quetiapine, cyamemazine, tricyclic antidepressants, and hypnotics) independently of weight gain and tobacco smoking status (23). These results are consistent with the increased CRP levels in SZ subjects of the phase 1 CATIE study who received 3 months of quetiapine treatment (28). However, in the last study, the results have not been adjusted for weight gain. Inconsistent results have been found for olanzapine (23, 28). In one observational study and one clinical trial, aripiprazole has been found to be associated with decreased CRP blood levels (23, 29). The exact mechanism of the association of some psychotropic drugs and increased or decreased inflammation is not fully understood to date. Clozapine has been found to be associated with increased inflammation through mitochondria alterations and insulin resistance (30). Aripiprazole has been previously associated with lower rates of metabolic syndrome (31) and metabolic syndrome is one of the major sources of inflammation in SZ subjects (2). Only one study assessed the biological effect of the administration of a single dose of aripiprazole (10 mg) on the pro-inflammatory cytokine IL-6 blood levels with negative results in healthy volunteers (32). A recent study has shown that aripiprazole can suppress inflammatory responses triggered by Gram positive bacteria through suppression of both AP-1 and NF-κB pathways (33). Ziprasidone was found to trigger a macrophage inflammatory response *in vitro* (34). Quetiapine has been associated with high rates of constipation (35) and constipation may be associated with microbiota dysbiosis (36, 37). It may therefore be hypothesized that quetiapine-induced inflammation may be induced by intestinal local inflammation that may increase the intestinal barrier permeability. A recent study has found abnormal translocation bacterial markers in peripheral blood of SZ patients (38). Altogether, these results suggest that quetiapine may be associated with pro-inflammatory disturbances in SZ patients. Microbiota and gut permeability analyses are needed in future studies to determine the mechanisms of quetiapine-induced inflammation.

Abnormal CRP levels have been associated with impaired quality of life in SZ in one study (15). No association between increased CRP and impaired functioning in schizophrenia has been reported to date.

### Cognition

Abnormal CRP has been associated with a various range of impaired cognitive functions in both in/acute and out/stabilized SZ individuals (14, 16, 39, 40). More specifically, increased CRP levels have been associated with impaired short-term memory (39), impaired general intellectual ability and abstract reasoning, working memory, memory, learning abilities, semantic memory, mental flexibility, visual attention and speed of processing (8).

### Physical health

Increased CRP levels have been associated with all-causes mortality in SZ (41) and 10-years cardio-vascular disease risk (42). A study comparing 483 SZ and BD subjects to HCs has concluded that Trauma-altered immune activation via elevated hs-CRP in patients with SZ and BD may be mediated by higher BMI (25).

Increased CRP levels have been extensively associated with increased BMI and therefore metabolic syndrome and cardiovascular risk (3, 13, 15, 16, 20, 24, 28, 42–50). Increased CRP has been associated with decreased vitamin D blood level (51, 52), higher sedentary behavior (53), and increased markers of intestinal bacterial translocation (38).

## Discussion

The literature has yielded inconsistent results in regard of the links between elevated CRP levels and the risk of later SZ onset. The results of the Prins et al. study, suggesting that CRP-associated alleles were associated with decreased risk of SZ (13) have fueled the debate about whether the CRP elevation in SZ is a by-product of the pathogenesis of SZ or directly contributing to clinical features of the disorder. These findings may also point out potential biases in previous studies regarding the causes of elevated CRP levels in SZ patients, such as pleiotropic effects within chosen instruments and/or reverse causality (13). In addition to CRP variants, other recent studies have identified other variants associated with SZ including variants in the major histocompatibility complex region on Chromosome 6p21 (54), harboring many cytokine genes (55–57), and in the TNF promoter (58), IL10 promoter (59), IL1B (60) and C4 (61). To make a long story short, CRP has been robustly associated with the SZ risk, however it remains unclear if this association may be due to confounding factors. This association was independent of BMI, and history of childhood trauma has not been associated with SZ risk to date. However, tobacco smoking, increased gut permeability (38), infections [especially Toxoplasma (62), HSV virus (63) or HERV-W endoretrovirus (64), candida albicans (65)], sleep disturbances, dental care and periodontal diseases (66) and impaired physical activity (53) may be all confounding factors for this association. It should be underlined that increased CRP has been associated with social withdrawal in the general population, social withdrawal being one the prodromal symptoms of schizophrenia in adolescents (67, 68).

The discrepancies between studies suggesting that peripheral inflammation is associated with positive symptoms and the others may be due to the psychotic phase status of the included patients (i.e., acute psychosis vs. stabilized/community-dwelling subjects). The studies that found no association between increased CRP and symptomatology have recruited community-dwelling stabilized outpatients (16, 22, 23).

One study has found that history of childhood trauma was associated with increased CRP in SZ adulthood (25), however this result has not been replicated in other studies (16) and this association disappeared after adjustment for BMI.

Inconsistent findings have been found in regard of the association between abnormal CRP, current depression and antidepressant consumption in SZ, with one study suggesting that increased CRP levels were associated with depressive symptoms, and one other that it was associated with antidepressant consumption (16, 24). This discrepancy may be due to different antidepressant administration, as the different classes of antidepressant have been associated with various anti-inflammatory properties (69).

Daily tobacco smoking is a major issue in SZ patients, more than half being current tobacco smokers (70). Increased CRP has been associated with high nicotine (NIC) dependence in SZ subjects. This finding was not consistent with the hypothesis that NIC dependence would be associated with lower peripheral inflammation due to the *in vitro* anti-inflammatory effects of nicotine (71, 72). Due to the cross-sectional design of the study, a causal relationship could not be drawn. The results of this study may support the self-medication hypothesis of tobacco smoking in SZ, which is still currently debated (73–76). As such, SZ smokers with increased CRP may self-administer nicotine to limit the negative effects of peripheral inflammation. The hypothesis of a genetic shared vulnerability between chronic peripheral inflammation and NIC dependence may also be suggested and has been described in other psychiatric disorders (77). As increased CRP and NIC dependence have both been associated with cognitive impairment in SZ (8, 78), it remains also to be determined if inflammation mediates the association between NIC dependence and cognitive impairment in SZ smokers. Preclinical and clinical studies have indicated that 7 nAChR deregulation may account for some of the cognition and mood SZ symptoms, with NIC use representing a strategy to alleviate these symptoms (79). It remain unclear to date if increased CRP levels at baseline may be associated with an increase rate of tobacco use relapse in tobacco cessation programs, and if NIC substitutes administration may improve peripheral inflammation in SZ patients.

Increased CRP levels have been associated with a wide range of impaired cognitive functions. While many studies [for meta-analysis see (80)] have suggested that anti-inflammatory add-on therapy may be effective in SZ subjects, no study has explored to date if adding anti-inflammatory agents to conventional treatment may improve cognitive function in SZ subjects with cognitive deficits and inflammatory disturbances. Anti-inflammatory strategies, combined with cognitive remediation therapy and benzodiazepine withdrawal when needed, may be the most effective personalized-medicine approach to improve cognition in SZ subjects (81).

The physical health studies have confirmed that increased CRP levels was a predictor of metabolic syndrome and cardiovascular risk in SZ subjects (42, 46, 48–50). Increased CRP have been associated with decreased 25-OH vitamin D levels, which may suggest that supplementing vitamin D may improve inflammatory status and cardio-vascular risk in SZ subjects with hypovitaminosis D (51, 52). As sedentarily behavior has been associated with increased CRP levels (53), physical activity may be suggested as the prior therapeutic intervention for SZ subjects with increased weight and peripheral inflammation. As translocation markers have been associated with increased CRP (38), interventions for restoring the intestinal barrier integrity (namely probiotics and diet interventions) may be useful to improve inflammation status in SZ subjects with microbiota disturbances/ increased gut permeability and peripheral low-grade inflammation.

### Limits

The risk of publication bias has been limited by the use of three databases, medline being considered as the database of reference with the highest quality studies, google scholar as the largest database, and web of science for exploring specific congress abstracts. Most of the included studies were cross-sectional. Because data on each participant are recorded only once it would be difficult to infer the temporal association between increased CRP and each explored outcome (82). Therefore, only an association, and not causation, can be inferred. These results may inform the hypotheses for a more complex investigation, such as a cohort study.

Some statistical approaches are commonly used to analyse CRP blood levels but they present some limits. The dichotomization of the variable using a cut-off raises the question of the (arbitrary) choice of this cut-off. No consensual cut-off values have been proposed in psychiatric studies for the analysis of CRP. A recent meta-analysis has pointed out that most of the included psychiatric studies used a cut-off ≥5 mg/L (3) while the international guidelines for predicting cardiovascular risk (“The Emerging Risk Factors Collaboration”; 2010) proposed a 3 mg/L cut-off. This last cut-off was also used in most of the psychiatric studies focusing on clinical symptoms and cognition. It remains unclear if these cut-offs, determined in non-psychiatric studies, are the most suitable for psychiatric studies. Moreover, the use of a dichotomized variable is questionable as it implies a loss of information (83). Considering CRP as a quantitative variable has led some researchers to use linear regression models. However, these models rely on the assumption of a normal distribution, which is not the case for CRP. It is possible to apply a log transformation of the data, which makes them more conform to normality (84). However, log transformation does not systematically help the data to be more normal or less variable (85, 86). Furthermore, log-transformed data cannot usually facilitate inferences concerning the original data, since it shares little in common with the original data (86). In the end, in the specific case of the CRP, the presence of a large number of patients with a value of 0 (undetectable) for the CRP makes this transformation impossible (0 values becoming –∞). In this context, the zero-inflated Poisson regression model may appear as the most suited statistical method, as it allows taking into account data which contain a substantial proportion of zero and with a highly skewed distribution, while keeping the whole of the information (87). This method has been used in only one study to date (23).

While sleep disorders have been suggested to have a bidirectional relationship with inflammation, no study has explored the relationships between abnormal CRP levels and sleep disorders in SZ to date. Except for antipsychotic effects, no longitudinal data has suggested if increased CRP levels were associated with poor prognosis and outcomes in schizophrenia (including hospitalizations, accelerated cognitive impairment and functioning). Further studies should explore if decreasing CRP blood levels may improve SZ outcomes, especially cardiometabolic events, tobacco smoking behavior, quality of life and cognitive functioning. As some add-on anti-inflammatory strategies have shown effectiveness in SZ symptomatology (88), each anti-inflammatory drug should be independently evaluated (especially omega 3 fatty acid and aspirin, which have been suggested to be effective in some SZ subgroups) (89, 90). As CRP is a global marker of inflammatory disturbances, the relationship between increased CRP and respectively oxidative stress disturbances and hormonal disturbances should also be explored and may lead to other therapeutic options, like N-acetyl-cysteine add-on administration (88).

In the light of the above-mentioned studies, increased hs-CRP may be reasonably suggested as a marker for SZ onset risk, as well as a risk factor for increased positive symptoms, cognitive impairment, hypovitaminosis D, microbiota disturbances, cardiovascular and metabolic syndrome risk in SZ subjects, and increased nicotine dependence in SZ smokers. In case of increased CRP levels, anti-inflammatory strategies (add-on anti-inflammatory drugs including aspirin and omega 3 fatty acids, vitamin D supplementation, physical activity, probiotics) should be further evaluated.

## Author contributions

GF and LB selected the studies, analyzed the major outcomes and wrote the manuscript. CL and PA reviewed the manuscript. All authors approved the final version.

### Conflict of interest statement

The authors declare that the research was conducted in the absence of any commercial or financial relationships that could be construed as a potential conflict of interest.
